# Beat Processing Is Pre-Attentive for Metrically Simple Rhythms with Clear Accents: An ERP Study

**DOI:** 10.1371/journal.pone.0097467

**Published:** 2014-05-28

**Authors:** Fleur L. Bouwer, Titia L. Van Zuijen, Henkjan Honing

**Affiliations:** 1 Institute for Logic, Language and Computation, University of Amsterdam, Amsterdam, The Netherlands; 2 Amsterdam Brain and Cognition (ABC), University of Amsterdam, Amsterdam, The Netherlands; 3 Research Institute of Child Development and Education, University of Amsterdam, Amsterdam, The Netherlands; ARC Centre of Excellence in Cognition and its Disorders (CCD), Australia

## Abstract

The perception of a regular beat is fundamental to music processing. Here we examine whether the detection of a regular beat is pre-attentive for metrically simple, acoustically varying stimuli using the mismatch negativity (MMN), an ERP response elicited by violations of acoustic regularity irrespective of whether subjects are attending to the stimuli. Both musicians and non-musicians were presented with a varying rhythm with a clear accent structure in which occasionally a sound was omitted. We compared the MMN response to the omission of identical sounds in different metrical positions. Most importantly, we found that omissions in strong metrical positions, on the beat, elicited higher amplitude MMN responses than omissions in weak metrical positions, not on the beat. This suggests that the detection of a beat is pre-attentive when highly beat inducing stimuli are used. No effects of musical expertise were found. Our results suggest that for metrically simple rhythms with clear accents beat processing does not require attention or musical expertise. In addition, we discuss how the use of acoustically varying stimuli may influence ERP results when studying beat processing.

## Introduction

In music, people often perceive regularly recurring salient events in time, known as the beat [1], [2]. Beat perception has been suggested to be a fundamental and innate human ability [3] and has been explained as neural resonance at the frequency of the beat [4]–[7] caused by regular fluctuations in attentional energy [8]. While the ease with which humans can pick up a beat is remarkable, it remains an open question how much attentional resources are needed to detect a beat. Some suggested that focused attention is necessary both for beat perception [9], [10] and regularity detection in general [11]. Others argued that beat processing and possibly even the processing of meter – alternating stronger and weaker beats – are in fact pre-attentive [12]–[14] and that beat processing might even be functional in (sleeping) newborns [15].

In the former studies, in which no evidence of beat processing without attention was found, only the temporal structure of the rhythm was varied to indicate the metrical structure [9] and highly syncopated rhythms were used [10]. Conversely, the latter studies [12], [15] used strictly metrical stimuli with not only variation in the temporal structure of the rhythm, but also variation in the timbre and intensity of tones to convey the metrical structure. The use of such acoustically rich, ecologically valid stimuli could be essential to allow the listener to induce a beat pre-attentively [14], arguably because multiple features in the stimuli carry information about the metrical structure. However, in these studies a beat was induced by using different sounds for metrically strong and metrically weak positions. While these different sounds may have aided in inducing a beat, this leaves open the possibility that different responses to tones in different metrical positions are due to acoustic differences rather than beat processing [16]. To rule out this explanation, in the current study, we test whether beat processing is pre-attentive using stimuli that resemble real music whilst probing positions varying in metrical salience but with identical acoustic properties.

We examine beat processing with a mismatch negativity (MMN) paradigm. The MMN is an auditory ERP component that is elicited when acoustic expectations are violated [17], [18]. The MMN is known to be independent of attention and the amplitude of the MMN response indexes the magnitude of the expectancy violation [19]. Also, the MMN response has been shown to correlate with behavioral and perceptual measures of deviance detection [19]–[22]. We compare the pre-attentive MMN response to unexpected omissions of sounds in different metrical positions in a music-like rhythm. As the omission of a sound in a metrically strong position is a bigger violation of the metrical expectations than the omission of a sound in a metrically weak position, we expect the MMN response to depend on the metrical position of the omissions, with larger responses for omissions in metrically stronger positions.

Finally, we compare the responses of musicians and non-musicians. Earlier, it has been shown that musical training affects beat processing [23] and can enhance several aspects of pre-attentive auditory processing, including melodic encoding [24], detection of numerical regularity [25] and sequence grouping [26]. Here we assess whether musical training can also affect the pre-attentive processing of temporal regularity. If beat processing is indeed a fundamental human ability, we expect to find no difference between musicians and non-musicians. However, if beat processing is learned behavior, we expect this ability to be influenced by musical expertise and thus we expect a bigger effect of metrical position on the MMN responses in musicians than in non-musicians.

## Materials and Methods

### Ethics Statement

All participants gave written informed consent before the study. The experiment was approved by the Ethics Committee of the Faculty of Social and Behavioral Sciences of the University of Amsterdam.

### Participants

Twenty-nine healthy adults participated in the experiment. Fourteen were professional musicians, or students enrolled in a music college (mean age, 29 years; age range, 22–57 years; 8 females). On average, they had received 18.5 years of musical training (range 9–36 years) and they reported playing their instrument at the time of the experiment on average 3.4 hours per day (range 1–5 hours). This group was considered musicians. Fifteen participants (mean age, 31 years; age range, 22–55 years; 9 females) did not play an instrument at the time of the experiment and had received on average 1.2 years of musical training (range 0–2 years), ending at least 10 years prior to the experiment. These participants were considered non-musicians. All participants had received college education or higher and none reported a history of neurological or hearing problems.

### Stimuli

**Figure 1:**
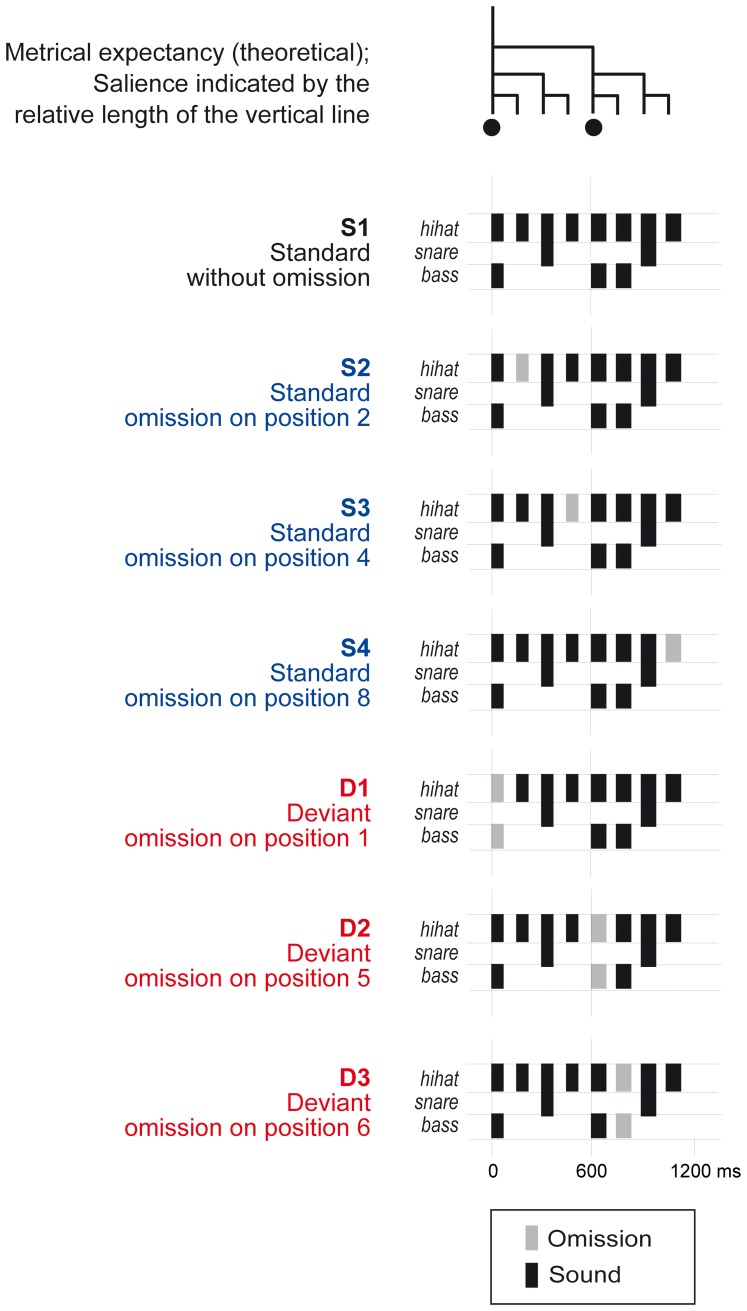
Schematic illustration of the rhythmic patterns used in the experiment. The pattern consisted of eight sounds and was designed to induce a rhythm with a hierarchical metrical structure (see tree-structure at the top; beats are marked with dots). The omissions occurred in positions varying in metrical salience, with the omissions in D1 on the first beat, the omissions in D2 on the second beat and the other omissions in equally weak metrical positions.

We presented participants with a continuous stream of varying rhythm designed to induce a regular beat in a music-like way (for studies using a similar paradigm, see [12], [15], [27]). We used a rhythmic sequence composed of seven different patterns. Of these patterns, four were used as standard patterns (S1–S4) and three were used as deviant patterns (D1–D3). Figure 1 shows an overview of all patterns. The base pattern (S1) consisted of eight consecutive sounds, with an inter-onset interval of 150 ms and a total length of 1200 ms. Hi-hat, snare drum and bass drum sounds were organised in a standard rock music configuration. We created sounds using QuickTime's drum timbres (Apple Inc.). The bass drum and snare drum sounds always occurred together with a simultaneous hi-hat sound. For the remainder of this paper, we will refer to these combined sounds as bass drum sound (positions one, five and six, see Fig. 1) and snare drum sound (positions three and seven, see Fig. 1). Sound durations were 50, 100 and 150 ms for hi-hat, bass drum and snare drum respectively.

**Figure 2:**
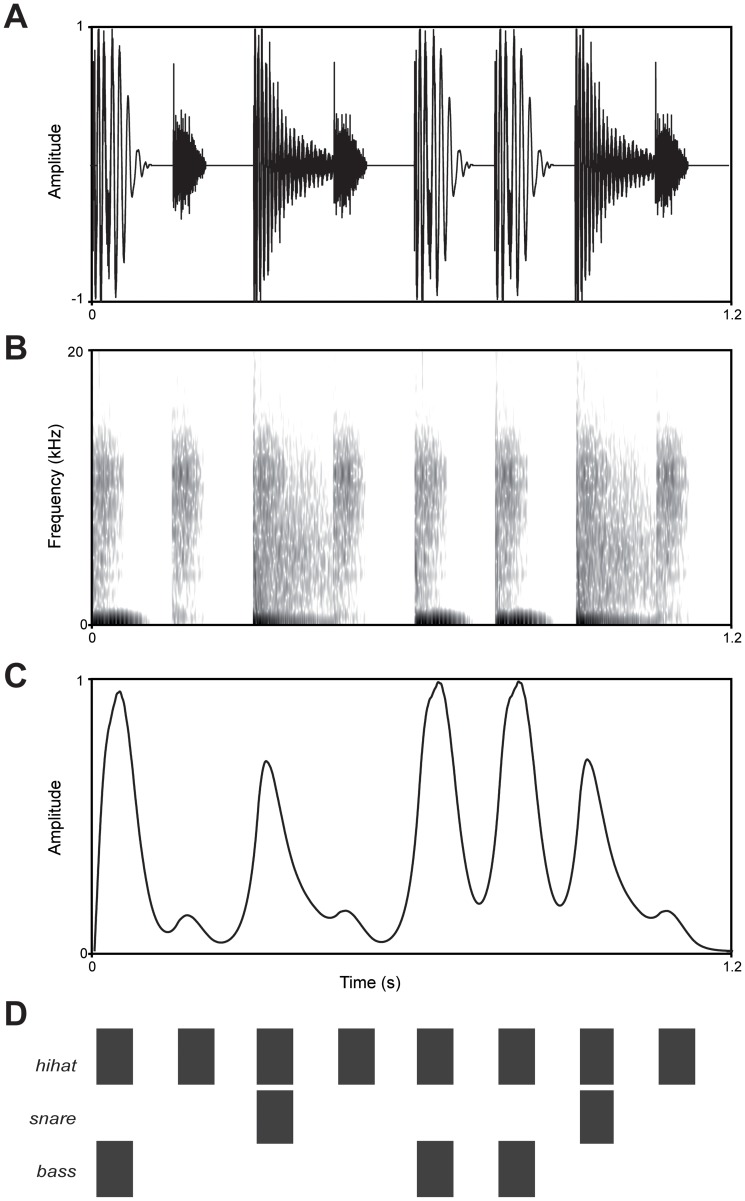
Acoustic analyses of stimulus S1. A) Waveform, B) spectrogram, C) amplitude envelope, and D) diagram of stimulus S1 (cf. Fig. 1). The spectrogram was calculated with a Short Time Fourier Transform, Gaussian window, window size 2 ms, time resolution 5 ms, frequency resolution 20 Hz, and 50 dB dynamic range. The amplitude envelope was calculated using a loudness model as described in [43].

Figure 2 depicts the acoustic properties of the base pattern (S1). The intensity of the bass drum sound was largest, followed by the intensity of the snare drum sound. The hi-hat sound had the lowest intensity. Therefore, the latter, the shortest and softest sound, would likely be interpreted as metrically weakest, while the bass drum sound would likely be interpreted as metrically strongest. This is in line with the way this pattern is often used in Western music, in which the bass drum indicates the downbeat, the snare drum indicates the offbeat and the hi-hat is used for subdivisions at the weakest metrical level. We expected the bass drum sounds at positions one and five to be interpreted as beats as they occurred with a regular inter-onset interval of 600 ms. As such, the pattern was expected to induce a beat at 100 beats per minute, a tempo close to the preferred rate for beat perception [28]. At this rate, each pattern encompassed two beats. The first and fifth position of the pattern coincided with respectively the first and second beat, while the second, fourth, sixth and eighth position were metrically weak positions (Fig. 1).

The base pattern (S1) was varied to create three additional standard patterns (S2–S4). In these patterns a hi-hat sound was omitted in positions two (S2), four (S3) and eight (S4). As such, the omissions in the standard patterns were all in metrically weak positions, that is, not on the beat. Together, the four standard patterns created a rhythm in which the surface structure varied, as is the case in natural music, but in which the metrical structure was left intact, to be maximally beat inducing. The standard patterns accounted for 90% of the total patterns.

The standard patterns were interspersed with three infrequent deviant patterns, accounting for the remaining 10% of the total patterns. In the deviant patterns (D1–D3) a bass drum sound was omitted. In deviant pattern D1 the sound on the first beat (position one), the most salient position in the pattern, was omitted. In deviant pattern D2 the sound on the second beat (position five) was omitted. Both in pattern D1 and in pattern D2 the omission of a sound on the beat violated the metrical structure and created a syncopation. In the third deviant pattern (D3), the same sound was omitted as in patterns D1 and D2, but in a metrically weak position (position six), leaving the metrical structure of the pattern intact.

We examined the presence of pre-attentive beat and meter processing by comparing the MMN responses to the omissions in the deviant patterns. We expected the magnitude of the MMN response to be affected by the metrical position of the omissions in two ways. First, we expected the amplitude of the MMN to omissions in D1 and D2, which were on the beat and thus violated the metrical expectations, to be larger than the amplitude of the MMN to omissions in D3, which was not on the beat and thus left the metrical structure intact. Such a difference would indicate that a beat was detected by the auditory system. Second, we expected to find a larger MMN response to omissions in D1 (on the first beat) than to omissions in D2 (on the second beat) as the former are bigger violations of the metrical expectations than the latter. Such a difference would suggest that a hierarchy between consecutive beats was detected, hence would be evidence for meter processing.

Importantly, the omissions in patterns D1, D2 and D3 could not be distinguished from each other based on the acoustic properties of the sound that was omitted (a bass drum sound) or their probability of occurrence (0.033 for each deviant pattern). Thus, we probed three metrically different positions with exactly the same procedure. Post hoc, we also assessed the effects of the acoustic variation in the stimuli by comparing the MMN responses to omissions of acoustically different sounds that were all in metrically equally weak positions, that is, the omissions in patterns D3 (a bass drum sound), S2, S3 and S4 (hi-hat sounds).

The patterns were delivered as a randomized continuous stream, without any gaps between consecutive patterns (see Sound S1 for a short example of the stimuli in a continuous stream). There were two constraints to the randomization. First, a deviant pattern was always preceded by at least three standard patterns. Second, no deviant pattern could be preceded by standard pattern S4, because this could potentially create two consecutive gaps. In the EEG experiment the stimuli were presented in 20 blocks of 300 patterns. Of these, 10% were deviant patterns, making the total number of trials for each of the three positions 200. Six additional standard patterns were added to the beginning (5) and end (1) of each block. Thus, each block lasted just over 6 minutes and the total number of standard patterns in the whole experiment was 5520, or 1380 trials for each of the four standard patterns. Stimuli were presented through two custom made speakers at 60 dB SPL using Presentation® software (Version 14.9, www.neurobs.com).

### Procedure

Participants were tested individually in a soundproof, electrically shielded room at the University of Amsterdam. During presentation of the sounds, they watched a self-selected, muted, subtitled movie on a laptop screen. Every block of stimuli was followed by a break of 30 seconds. Longer breaks were inserted at the participants' need. Participants were instructed to ignore the sounds and focus on the movie. In a questionnaire administered after the experiment all of the participants reported being able to adhere to these instructions. This questionnaire was also used to obtain information about their musical experience. Including breaks, the entire experiment took around 2,5 hours to complete.

### EEG recording

The EEG was recorded with a 64 channel Biosemi Active-Two reference-free EEG system (Biosemi, Amsterdam, The Netherlands). The electrodes were mounted on an elastic head cap and positioned according to the 10/20 system. Additional electrodes were placed at the left and right mastoids, on the tip of the nose and around the eyes to monitor eye movements. The signals were recorded at a sampling rate of 8 kHz.

### EEG analysis

EEG pre-processing was performed using Matlab (Mathworks, Inc.) and EEGLAB [29]. The EEG data was offline re-referenced to linked mastoids, down-sampled to 256 Hz and filtered using 0.5 Hz high-pass and 20 Hz low-pass FIR filters. For seven participants, one bad channel was removed and replaced by values interpolated from the surrounding channels. None of these channels is included in the statistical analysis reported here. Independent component analysis as implemented in EEGLAB was conducted to remove eye blinks. For the deviant patterns (D1–D3) and the three standard patterns containing omissions (S2–S4), epochs of 800 ms were extracted from the continuous data starting 200 ms before the onset of the omission. Epochs with an amplitude change of more than 75 µV in a 500 ms window on any channel were rejected. Finally, epochs were baseline corrected by the average voltage of the 200 ms prior to the onset of the omission and averaged to obtain ERPs for omissions in each position for each participant.

The omissions in the various patterns could be preceded by a bass drum sound (D3 and S2), a snare drum sound (S3 and S4) or a hi-hat sound (D1 and D2). To control for the possible effects of this contextual difference we calculated difference waves. For all patterns containing omissions, from the ERP obtained in response to the omissions we subtracted the temporally aligned ERP obtained from base pattern S1. This procedure yielded difference waves for each participant that were thought to reflect only the additional activity elicited by the omission in that particular position.

Visual inspection of the group averaged difference waves showed negative deflections peaking between 100 and 200 ms after the onset of each omission with a frontocentral maximum. This is consistent with the latency and scalp distribution of the MMN [19]. Hence, MMN latencies were subsequently defined as the negative peak on electrode FCz between 100 and 200 ms. Single subject amplitudes were defined for each condition as the average amplitude in a 60 ms window around the condition specific peaks obtained from the group averaged difference waves.

The group averaged difference waves also showed positive deflections consistent in latency and scalp distribution with a P3a [30]. However, in the latency range of the P3a the ERPs could possibly contain contributions from activity related to the tone following the omission, which occurred 150 ms after the omission. While the use of difference waves might eliminate some of this activity, the tones following an omission could possibly elicit an enhanced N1 response due to fresh afferent neuronal activity. This additional activity may be absent in the ERPs for S1, which we used to obtain the difference waves and thus would not be eliminated by the subtraction procedure. Due to the different sounds following the omissions in the deviants (Fig. 1), such an effect would be different for each deviant. Differences between the ERPs in the latency range of the P3a are thus hard to interpret. Therefore, here we will only consider the MMN results.

### Statistical analysis

To confirm that the MMN peaks were significantly different from zero, we performed T-tests on the MMN amplitudes for each condition separately on electrode FCz. Our primary interest concerned the difference in response to omissions in the deviant patterns, to evaluate the effects of metrical position and musical expertise. Thus, first we compared the amplitude and latency of the MMN response to the omissions in the deviant patterns in a repeated measures ANOVAs, with position (D1, D2, D3) as a within subject factor and musical expertise (musician, non-musician) as a between subject factor. In addition, to examine the effects of using acoustically varying stimuli we compared the MMN responses to omissions in D3, S2, S3 and S4 in ANOVAs with the same structure. Greenhouse-Geisser corrections were used when the assumption of sphericity was violated. For significant main effects, Bonferroni-corrected post hoc pairwise comparisons were performed. The statistical analysis was conducted in SPSS (Version 20.0). We report all effects that are significant at *p*<0.05.

## Results

Table 1 shows the average mean amplitudes and peak latencies of the MMN for omissions in all patterns. T-tests confirmed that the amplitudes of the negative peaks in the difference waves between 100 and 200 ms from the onset of the omissions were significantly different from zero for both musicians and non-musicians and for omissions in all positions (all *p* values <0.001), showing that an MMN was elicited by all omissions.

**Table 1:** Mean average amplitudes and average peak latencies of the MMN to omissions in all conditions.

	Average Amplitude (µV)		Average Peak Latency (ms)	
	Musicians (N = 14)	Non-musicians (N = 15)	Musicians (N = 14)	Non-musicians (N = 15)
D1	−3.49 (1.43)	−3.70 (1.96)	146 (22)	142 (19)
D2	−3.12 (1.18)	−3.26 (1.73)	144 (16)	148 (16)
D3	−2.05 (1.26)	−2.38 (1.14)	129 (21)	117 (17)
S2	−1.55 (0.64)	−1.64 (0.86)	136 (17)	135 (19)
S3	−1.09 (0.69)	−.97 (0.79)	151 (33)	157 (37)
S4	−1.15 (0.75)	−1.03 (0.76)	136 (28)	157 (31)

*Note.* Standard deviations in brackets.

### Response to omissions in deviant patterns

**Figure 3:**
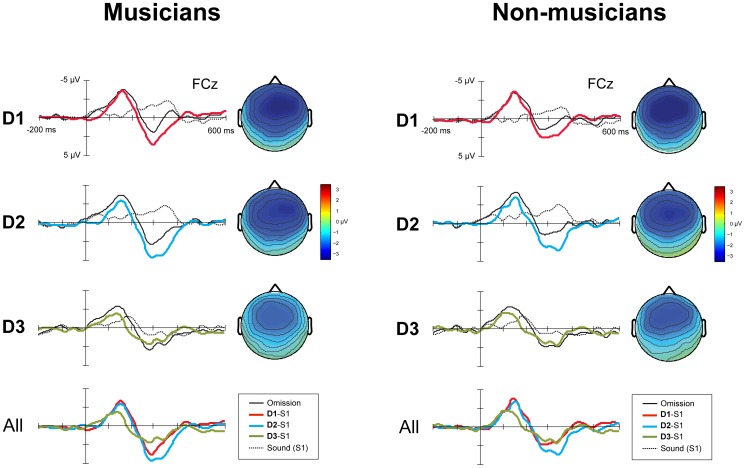
ERP responses for D1, D2 and D3 for musicians (N = 14, left) and non-musicians (N = 15, right). The panels labeled D1, D2 and D3 show the group averaged ERPs for electrode FCz elicited by omissions, the corresponding position in S1, the derived difference waves and the scalp distributions of the difference waves. The panel labeled *All* shows all difference waves combined. Time 0 is the onset of the omission, or, in the case of S1, the onset of the corresponding sound. The omissions in D1, D2 and D3 were equally rare in occurrence (0.033) and in all cases, a bass drum sound was omitted.

Figure 3 shows the group averaged ERPs and difference waves for omissions in the three deviant patterns (D1, D2 and D3) for electrode FCz for both musicians and non-musicians. The position of the omissions in the deviant patterns had a significant effect on both the amplitude (*F*_(2,54)_  = 19.4, *p*<0.001, *η^2^* = 0.42) and the latency (*F*_(2,54)_  = 24.0, *p*<0.001, *η^2^* = 0.47) of the MMN. Post hoc pairwise comparisons revealed that this was due to the MMN to the omissions in D3 being smaller in amplitude and earlier in latency than the MMN to the omissions in both D1 and D2 (all *p* values <0.001). The amplitudes of the responses to omissions in D1 and D2 did not differ from each other (amplitude, *p* = 0.191; latency, *p* = 1.000). Neither the effect of musical expertise (amplitude, *F*_(1,27)_  = 0.21, *p* = 0.647, *η^2^* = 0.008; latency, *F*_(1,27)_  = 0.42, *p* = 0.521, *η^2^* = 0.015) nor the interaction between musical expertise and position (amplitude, *F*_(2,54)_  = 0.09, *p* = 0.911, *η^2^* = 0.003; latency, *F*_(2,54)_  = 2.37, *p* = 0.103, *η^2^* = 0.081) was significant.

### Response to omissions in metrically weak positions

**Figure 4:**
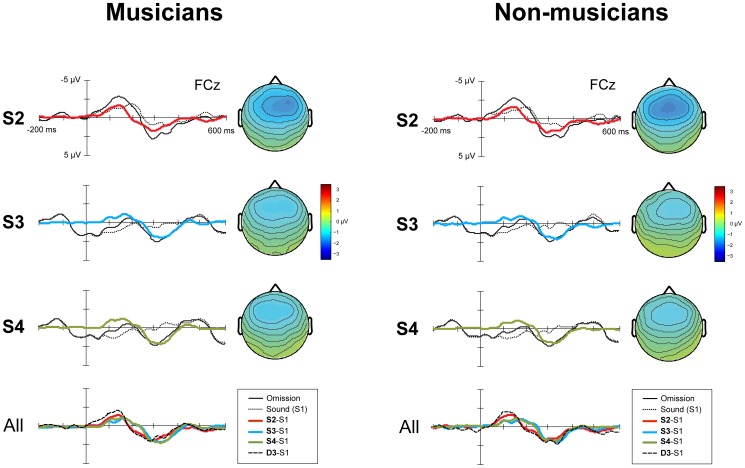
ERP responses for S2, S3 and S4 for musicians (N = 14, left) and non-musicians (N = 15, right). The panels labeled S2, S3 and S4 show the group averaged ERPs for electrode FCz elicited by omissions in the standards, the corresponding position in S1, the derived difference waves and the scalp distributions of the difference waves. The panel labeled *All* shows all difference waves combined. Time 0 is the onset of the omission, or, in the case of S1, the onset of the corresponding sound. The omissions in S2, S3 and S4 were equally rare in occurrence (0.225) and in all cases, a hi-hat sound was omitted. For clarity, here we add the difference wave for D3 (see Figure?3for the separate ERPs) to make a comparison with the difference waves derived for the standards possible. The omissions in D3 were in equally weak metrical positions as in S2, S3 and S4.

Figure 4 shows the ERPs elicited by all omissions in metrically weak positions (in patterns D3, S2, S3 and S4). The amplitude and latency of the MMN were significantly affected by the position of the omissions (amplitude, *F*_(3,81)_  = 25.4, *p*<0.001, *η^2^* = 0.48; latency, *F*_(3,81)_  = 9.99, *p*<0.001, *η^2^* = 0.27) but not by the factor musical expertise (amplitude, *F*_(1,27)_  = 0.03, *p* = 0.864, *η^2^* = 0.001; latency, *F*_(1,27)_  = 0.31, *p* = 0.580, *η^2^* = 0.012) or an interaction between musical expertise and position (amplitude, *F*_(3,81)_  = 0.96, *p* = 0.415, *η^2^* = 0.034; latency, *F*_(3,81)_  = 2.37, *p* = 0.077, *η^2^* = 0.081).

Post hoc pairwise comparisons revealed that the significant effect of position on MMN amplitude was due to the MMN to omissions in D3 being larger in amplitude than the MMN to omissions in S2 (*p = *0.002), S3 (*p*<0.001) and S4 (*p*<0.001). Interestingly, the amplitude of the MMN to the omissions in standard S2 was significantly larger than the amplitude of the MMN to the omissions in standards S3 (*p* = 0.005) and S4 (*p* = 0.011). Finally, the MMN to omissions in D3 was earlier in latency than the MMN to omissions in S2 (*p* = 0.040), S3 (*p* = 0.001) and S4 (*p* = 0.001).

## Discussion

The data show that the MMN responses to omissions on the beat (D1, D2) were larger in amplitude than the MMN response to omissions in a metrically weak position (D3), indicating that the former, which violated the metrical structure, were processed as more salient than the latter, which left the metrical structure intact (Fig. 3). The omissions could not be differentiated from each other based on their acoustic characteristics, suggesting that auditory system of the participants detected the beat pre-attentively.

Each pattern encompassed two beats. To examine whether participants detected a hierarchy between the two beats, we compared the MMN responses to omissions on the first (D1) and second (D2) beat (Fig. 3). We found no differences in amplitude or latency, suggesting that processing of meter – higher order regularity in the form of alternating stronger and weaker beats – is not pre-attentive. However, while the lack of an effect of the position of the beat may be indicative of a true absence of meter perception, two caveats must be noted. First, the MMN amplitude for omissions in both D1 and D2 was very large (<−3 µV) and maybe near ceiling, as it might contain the additive effects of multiple regularity violations, not only violations of the metrical structure, but also violations of the acoustic regularity (see below). This may have caused the tendency towards larger amplitude responses to D1 than D2, present in both musicians and non-musicians, not to reach significance. Second, while we assumed that the pattern was perceived as two consecutive beats, with D1 containing an omission on the first beat and D2 containing an omission on the second beat, the patterns in fact did not contain any accents indicating a hierarchy between a first and second beat. Therefore, it is possible that some participants processed the fifth position in the pattern as the first beat and the first position as the second beat. To address these issues and to examine meter processing, a paradigm more specifically tuned to inducing and measuring a hierarchy between beats is needed.

The MMN responses of musicians and non-musicians did not differ (Fig. 3; Table 1). Thus, not only may beat processing not require attention, but also it may be independent of musical expertise. Our findings are in contrast with earlier studies proposing a role for both attention [9], [10] and expertise [31] in beat processing. These conclusions were based on experiments in which the beat was marked only by temporal variation in the surface structure of the rhythm. In the current study, acoustically more varied stimuli were used, in which the beat was marked by both the surface structure of the rhythm and timbre and intensity differences. Arguably, the additional information contained in the acoustic properties of the sounds may make it easier to induce a beat, as accents are simply indicated by intensity differences and do not have to be deduced from the temporal organization of the rhythm. Therefore, we propose that conflicting findings regarding the role of attention and musical expertise in beat processing may be explained by looking at the temporal and acoustic complexity of the musical stimuli.

This view is further supported by studies suggesting that the use of real music leads to bigger effects of beat processing than the use of more abstract sequences of tones [14], [32], which may also be attributable to the real music containing multiple clues for the metrical structure. Finally, in a study directly comparing beat processing with only temporal accents and beat processing with only intensity accents it was suggested that the latter required less internal effort than the former [33]. Together with our results, these findings stress the importance of using more acoustically varied stimuli when testing beat processing. The use of highly abstract sequences of tones, with only variation in the temporal organization of the rhythm, may result in an underestimation of the beat processing abilities of untrained individuals.

While attention and expertise did not seem to affect beat processing with the current, highly beat inducing stimuli, we cannot rule out that beat processing, especially when more complex stimuli are used, is mediated to some extent by attention and expertise. However, our results support the view that for metrically simple, acoustically varied music-like rhythms, beat processing is possible without attention or expertise and may indeed be considered a very fundamental human ability [3].

To examine, exploratory, possible effects of acoustically rich stimuli on ERPs we compared the responses to omissions that varied acoustically but were all in metrically equally weak positions. As in each pattern only one out of eight tones was omitted, all these omissions could be considered rare events within a pattern, and as such, elicited an MMN (Fig. 4). The comparison between these MMN responses yielded two interesting effects. First, the MMN to omissions in pattern D3 was larger in amplitude than the MMN to omissions in the standard patterns (S2, S3 and S4). As it is known that low probability events cause higher amplitude MMN responses [34], this was presumably due to the omission of a bass drum sound, as in D3, being more rare than the omission of a hi-hat sound, as in S2, S3 and S4. Interestingly, to detect this probability difference, not only acoustic information but also information about the sequential order of the sounds is required. Thus, the auditory system formed a representation at the level of the complete pattern. This is consistent with the view that patterns as long as 4 seconds can be represented as a whole by the MMN system, whilst this system can operate at multiple hierarchical levels, representing both patterns and sounds within patterns simultaneously [35].

Second, unexpectedly, the amplitude of the MMN to omissions in S2 was larger than the amplitude of the MMN to omissions in S3 and S4 (Fig. 4). These omissions were all in metrically weak positions and in all cases a hi-hat sound was omitted. However, in S2, the omissions followed a bass drum sound, while in S3 and S4 the omissions followed a snare drum sound (Fig. 1). While we used difference waves to eliminate any direct effects of the acoustic context on the waveforms, the sounds preceding the omissions may have affected the MMN response indirectly by affecting the regularity representation [36] through forward masking [37]. Forward masking decreases with an increasing interval between the masking sound and the masked sound, the masker-signal delay [38]. Thus, the hi-hat sounds in positions four and eight, which immediately followed the snare drum sound with a delay of 0 ms, may have been perceptually less loud than the hi-hat sound in position two, which followed the bass drum sound with a delay of 50 ms. The omission of the former, in S3 and S4, may therefore have been perceived as acoustically less salient than the omission of the latter, in S2, explaining the difference in MMN amplitude.

The presence of this effect could potentially weaken our conclusions regarding pre-attentive beat processing, as the acoustic context of the omissions in D1 and D2, following a hi-hat sound with a delay of 100 ms, differed from the acoustic context of the omissions in D3, following a bass drum sound with a delay of 50 ms. However, it has been shown that increases in masker-signal delay affect the magnitude of masking nonlinearly, with more rapid decreases in masking at smaller masker-signal delays than at larger masker-signal delays [38], [39]. Therefore, any effect of masking on the MMN responses to omissions in D1, D2 and D3, with delays from 50 to 100 ms, should be the same or smaller than the effect of masking on the MMN responses to omissions in S2, S3 and S4, with delays from 0 to 50 ms. Yet the difference between the MMN responses to omissions in D3 and in D1 and D2 was much larger than the difference between the MMN responses to omissions in S2 and in S3 and S4. Consequently, contextual differences alone are unlikely to account for the difference between the response to omissions on the beat (D1 and D2) and omissions in metrically weak positions (D3).

To summarize, the differences in the responses to acoustically varying omissions in metrically weak positions show how the same sound differences that allow people to perceive a beat can cause difficulty in the interpretation of ERP results. Here, we controlled for these acoustic differences and show that adults differentiate pre-attentively between omissions in different metrical positions, based solely on their position. However, our results suggest that some caution has to be taken in interpreting earlier results in newborns [15]. It is unclear whether newborns, like adults in the current study, detected the beat solely based on its position in the rhythm. While not in conflict with these previous findings [15], our results do suggest the need for additional testing to fully confirm their conclusions.

The use of acoustically rich stimuli can be advantageous when testing beat processing [14], [32]. One way of addressing the possible pitfalls associated with such stimuli is by improving stimulus design, as in the current study. Alternatively, beat processing can be probed with alternative methods, which perhaps are less sensitive to acoustic factors than ERPs. Promising results have been obtained by looking at neural dynamics [40], [7] and steady-state potentials [5], [6], but so far only using simple isochronous or highly repetitive sequences. Combining these methods with acoustically rich and temporally varied stimuli may provide valuable information about beat processing and warrants further research.

## Conclusions

We have provided evidence suggesting that beat processing with metrically simple and acoustically varied stimuli does not require attention or musical expertise. Furthermore, we have shown that the MMN response to omissions in a rhythm is indeed sensitive to metrical position and as such can be a useful tool in probing beat processing, even if acoustically varied stimuli are used. Our conclusions are in line with previous findings in adults [12], [13] and newborns [15]. However, we also showed that the ability of the listener to recognize longer patterns and the acoustic context of an omission can influence the ERP response to sound omissions in a rhythm. While the present results are not in conflict with previous findings, controls for these issues were lacking in earlier experiments [12], [13], [15], [27]. To be certain that any effects observed are due to metrical position and not pattern matching or acoustic variability, future experiments will have to take these factors into account. At the same time, if sufficiently controlled, the use of stimuli with acoustic variability may be a big advantage when testing beat processing.

The current study thus not only contributes to the growing knowledge on the functioning of beat processing, it also nuances findings that were novel and exciting, but that are in need of additional testing to be fully confirmed. As such, the current study fits in a general trend that stresses the importance of replication in psychological research [41], [42].

## Supporting Information

Sound S1 **Example of the stimuli in a continuous stream.** In this example, each deviant appears once and in total 30 patterns have been concatenated. The order of appearance of the stimuli in this example is: S1-S4-S3-S1-S2-S1-S2-**D2**-S4-S2-S3-S2-S3-S3-S4-S1-S3-**D3**-S1-S4-S1-S2-S1-**D1**-S2-S4-S3-S4-S2-S4.
(WAV)
